# In vivo γ‐aminobutyric acid measurement in rats with spectral editing at 4.7T

**DOI:** 10.1002/jmri.25093

**Published:** 2015-12-03

**Authors:** Stephen J. Sawiak, Bianca Jupp, Tom Taylor, Daniele Caprioli, T. Adrian Carpenter, Jeffrey W. Dalley

**Affiliations:** ^1^ Behavioural and Clinical Neuroscience Institute, University of CambridgeUK; ^2^ Wolfson Brain Imaging Centre, University of CambridgeUK; ^3^ Cavendish Laboratory, University of CambridgeUK; ^4^ Department of PsychologyUniversity of CambridgeUK; ^5^ Department of PsychiatryUniversity of CambridgeUK

**Keywords:** spectroscopy, behavior, nucleus accumbens, striatum, GABA

## Abstract

**Purpose:**

To evaluate the feasibility of spectral editing for quantification of γ‐aminobutyric acid (GABA) in the rat brain and to determine whether altered GABA concentration in the ventral striatum is a neural endophenotype associated with trait‐like impulsive behavior.

**Materials and Methods:**

Spectra were acquired at 4.7T for 23 male Lister‐hooded rats that had been previously screened for extremely low and high impulsivity phenotypes on an automated behavioral task (*n* = 11 low‐impulsive; *n* = 12 high‐impulsive). Voxels of 3 × 7 × 4 mm^3^ (84 μL) centered bilaterally across the ventral striatum were used to evaluate GABA concentration ratios.

**Results:**

Quantifiable GABA signals in the ventral striatum were obtained for all rats. Mean‐edited GABA to n‐acetyl aspartate (NAA) ratios in the ventral striatum were 0.22 (95% confidence interval [CI] [0.18, 0.25]). Mean GABA/NAA ratios in this region were significantly decreased by 28% in high‐impulsive rats compared to low‐impulsive rats (*P* = 0.02; 95% CI [–53%, –2%]).

**Conclusion:**

These findings demonstrate that spectral editing at 4.7T is a feasible method to assess in vivo GABA concentrations in the rat brain. The results show that diminished GABA content in the ventral striatum may be a neural endophenotype associated with impulsivity. J. Magn. Reson. Imaging 2016;43:1308–1312.

Gamma‐aminobutyric acid (GABA) is a key amino acid in the brain mediating fast inhibitory neurotransmission.^1^ Perturbation in GABAergic transmission has been widely implicated in epilepsy and other brain disorders.^2^, ^3^, ^4^ However, the quantification of GABA by noninvasive magnetic resonance spectroscopy (MRS) is hampered by its low concentration in the brain and overlapping signals from other metabolites, notably creatine at 3.0 ppm with an ∼10‐fold greater abundance in brain tissue.^5^

Efforts to measure GABA in vivo have included short echo time sequences, 2D *J*‐resolved spectroscopy, and spectral editing sequences, the most widely used of which is Mescher‐Garwood point‐resolved spectroscopy, or MEGA‐PRESS.^5^, ^6^ The ^1^H spectrum of GABA (H_3_N‐CH_2_‐CH_2_‐CH_2_‐COO^‐^) has three multiplets at ∼1.9 ppm, 2.3 ppm, and 3.0 ppm, which overlap with n‐acetylaspartate (NAA), glutamine/glutamate, and creatine, respectively. In MEGA‐PRESS, selective saturation of the GABA methylene protons at 1.9 ppm affects the coupled GABA spins at 3 ppm, while the uncoupled creatine resonance at 3 ppm is not affected by editing. Thus, subtracting the unedited from the edited spectrum yields a GABA resonance without overlapping creatine signal. For details of MEGA‐PRESS acquisition, see Refs. 6, 7, 8.

The present study investigated the feasibility and utility of MEGA‐PRESS at 4.7T for resolving regional GABA content in the rat brain.

## Materials and Methods

### Behavioral Screening

Male Lister‐hooded rats (*n* = 96) were screened for impulsivity on the five choice serial reaction time task (5‐CSRTT).^9^ Briefly, rats were trained in an operant chamber (Med Associates, USA) to detect the location of brief (700 msec), spatially unpredictable, visual stimuli presented on a discrete trial basis in one of five open recesses.^10^ If the rat selected the correctly illuminated recess, a 45 mg food pellet was delivered to a magazine in the chamber. If the rat did not respond or selected an incorrect opening, the chamber lights were switched off for 5 seconds and no food pellet was dispensed. A premature response (ie, a response made before the onset of the imperative signal to respond) triggered the same outcome as an incorrect response, with no food pellet delivered on that trial. Once rats had acquired the task over ∼4 months of training, they were screened for impulsivity over a 3‐week period. Rats underwent 100 trials in the apparatus on days 1, 2, 4, and 5, with an intertrial interval (the interval marking the start of the trial and onset of the light stimulus) of 5 seconds. On day 3 the intertrial interval was extended to 7 seconds as a deliberate challenge to increase the number of premature responses.^11^ Mean rates of premature responses over the three challenge sessions were used to define rats as either highly impulsive (a mean number of premature responses ≥50) or weakly impulsive (average premature responses ≤30). The 12 rats with the highest rates of premature responses and 12 rats with the lowest rates of premature responses were selected as “high impulsivity (HI)” and “low impulsivity (LI)” groups, respectively.

All experimental procedures were reviewed and approved by a local Ethical Review Committee and were conducted in compliance with appropriate regulatory requirements.

### MEGA‐PRESS Acquisition

A series of in vitro samples containing GABA (Sigma Aldrich, St. Louis, MO) in distilled water (0.5, 1.0, 2.0, 5.0, and 10 mM) were scanned to confirm that our implementation of the sequence was correct and gave values corresponding to known concentrations.

Rats were anesthetized with isoflurane (1.5–2.5% in 1L/min medical O_2_). For physiological monitoring, respiration rates were measured with a pressure sensor, body temperature was measured with a rectal probe, and oxygen saturation with pulse oximetry (SA Instruments, New York, NY). Anesthetic dose rates were adjusted to keep respiration rates within an appropriate physiological range and a flowing water heating pad was used to maintain body temperature throughout the scan.

A Bruker BioSpec 47/40 4.7T system with ParaVision 5.1 (Bruker, Germany) was used with a 72 mm birdcage transmit coil (Bruker model T2000) and manufacturer‐provided four‐channel receive array for reception (Bruker model T13014). For placement of the voxel, localizer and rapid acquisition with relaxation enhancement images were acquired (repetition time 15.3 sec, effective echo time 36 msec, echo train length 8, field of view 64 × 64 mm^2^, matrix 256 × 256 for 250 μm planar resolution with 250 μm slice thickness in 6 min 7 sec).

MEGA pulses were applied at offsets of 1.9 ppm and 7.5 ppm on alternate acquisitions, based on the MEGA‐PRESS sequence with TE1 = 12 msec and TE2 = 56 msec. Gaussian pulses of 20 msec duration were used. Spoiler gradients of 370 mT/m were applied for 1 msec in the scheme proposed previously.^6^ Water suppression was manually adjusted for each subject using variable power and optimized relaxation delays (VAPOR).^12^ The manufacturer‐provided map shim method was used to adjust shim currents calculated from a measured field map.

The repetition time was 2 seconds and echo time was 68 msec. For acquisition, 2048 points were acquired in 511 msec (bandwidth 4 kHz corresponding to 20 ppm). A total of 256 transients were obtained (128 “on” and 128 “off”) for an acquisition time of 8 minutes 32 seconds per animal. Water reference scans were acquired in a single shot for reference prior to each acquisition to calibrate the phase of the array coil elements.

### Spectral Processing and Analysis

Raw data were processed in MatLab 2011b (MathWorks, Natick, MA). A circular shift of 68 points was applied to the raw data to compensate for the digitization group delay and data were zero‐filled to 8192 points with line‐broadening of 3 Hz. To correct frequency and phase drift between transients and identify corruption by motion or physiological disturbance, the creatine peak from each transient was fitted and the parameters used to correct or reject entirely the transient. This was done by first calculating mean “on” and “off” spectra to obtain the mean values of frequency and linewidth for the creatine peak at 3.0 ppm. These values were then used to initialize linear least‐squares fitting for the same parameters in each transient separately. Transients were excluded if the frequency of the peak fitted at 3.0 ppm differed by more than 4 SD (standard deviation) of the median value, or where the full width half maximum or phase differed by more than 2 SD of their median values, respectively.

The fitted Lorentzian parameters were used to adjust the frequency and phase of each transient before averaging to produce mean “on” and “off” spectra. To obtain GABA/NAA ratios, the NAA peak at 2.0 ppm was fitted to a Lorentzian function and the GABA peak at 3.0 ppm was fitted to a single Gaussian function.^13^

### Statistical Analysis

The fitted Lorentzian peak areas were compared between HI and LI animals using Student's *t*‐test. One‐sided tests were used to compare the null hypothesis with the a priori hypothesis that HI rats would show reduced GABA ratios compared to LI rats with *P* < 0.05 used as the threshold for statistical significance.

## Results

**Figure 1:**
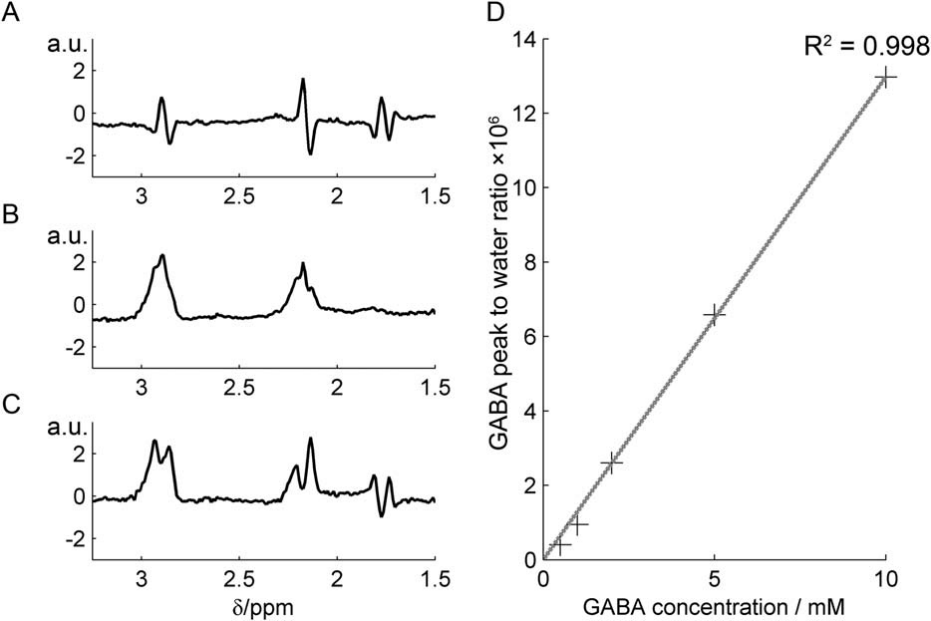
Typical GABA solution spectra for MEGA‐PRESS “off” **(A)**, MEGA‐PRESS “on” **(B)**, and difference spectrum **(C)**. Plot in **(D)** shows the ratio of the GABA peak area at 3 ppm to the unsuppressed water peak against prepared concentration showing good agreement.

**Figure 2:**
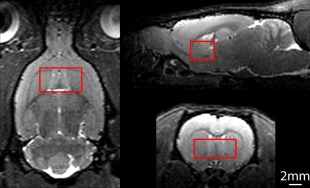
Typical voxel placement for the MEGA‐PRESS acquisition showing horizontal, sagittal, and coronal sections, clockwise from left.

**Figure 3:**
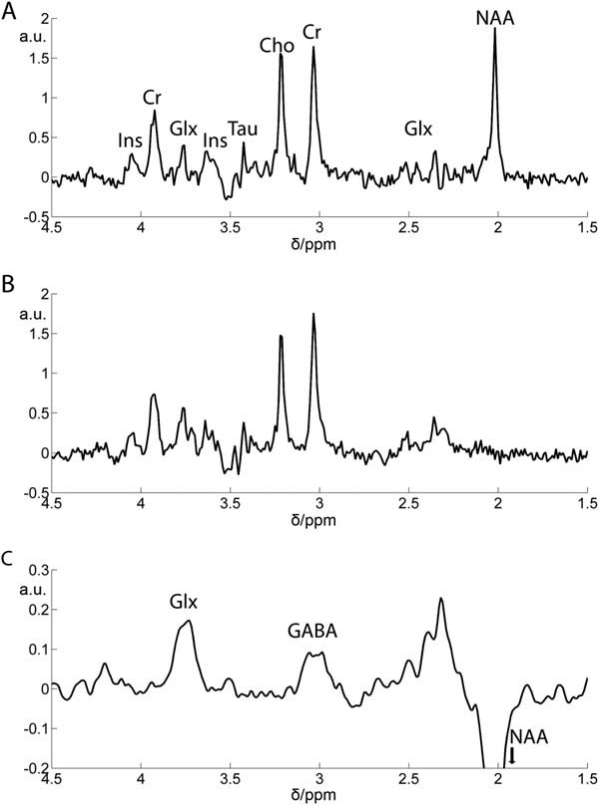
Typical spectra seen for MEGA‐PRESS “off” **(A)**, MEGA‐PRESS “on” **(B)** and the edited spectrum **(C)**. Note the expanded scale in (C). Line broadening of 7.5 Hz has been applied in (C) for clarity. Major peaks in (A) are labeled as Ins (inositol), glutamine/glutamate (Glx), taurine (Tau), choline (Cho), creatine, and phosphocreatine (Cr).

In vitro results are shown in Fig. 1. Good agreement was observed between prepared GABA standards and the signal measured (R^2^ = 0.998) and the spectra “on,” “off,” and edited difference spectra have the expected form. Illustrative voxel placement is shown in Fig. 2, with in vivo acquisition spectra shown in Fig. 3. For clearer presentation, line broadening of 7.5 Hz was applied to the edited spectrum and is shown with an expanded scale. One LI rat did not recover from anesthesia. As a precaution, data from that subject were not processed or considered further in the analysis.

**Figure 4:**
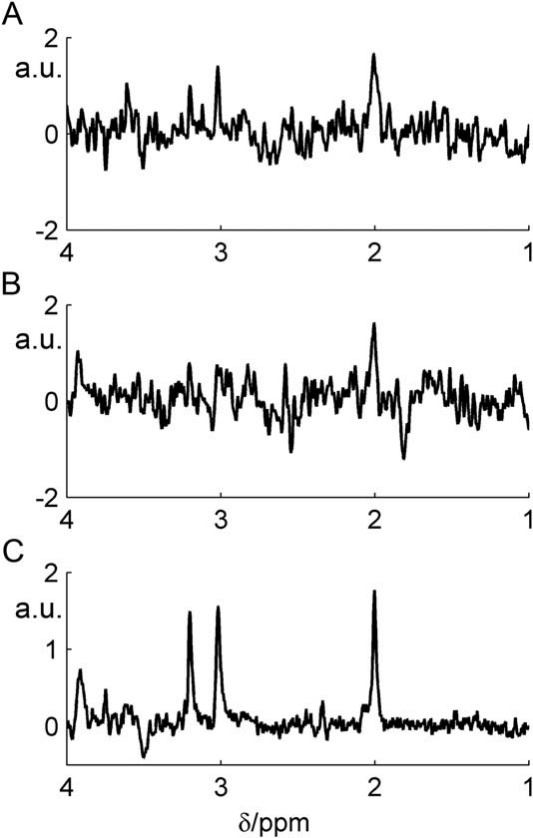
Illustrative accepted transient **(A)**, rejected transient **(B)**, and the mean spectrum from a typical subject for comparison (MEGAPRESS “off”).

Good shimming was achieved for all rats with unsuppressed water linewidths below 10 Hz (the mean ± SD across all measured spectra here was 7.5 ± 1.1 Hz, full‐width half‐maximum). On average, 13 transients were discarded from each series (SD 2.1) due to frequency drifts, excessive linewidth or phase shifts. Typical frequency drift was 3 Hz (0.015 ppm). Figure 4 shows typical single transients that were accepted (A), rejected (B) and the mean MEGA‐PRESS “off” spectrum for comparison (C).

**Figure 5:**
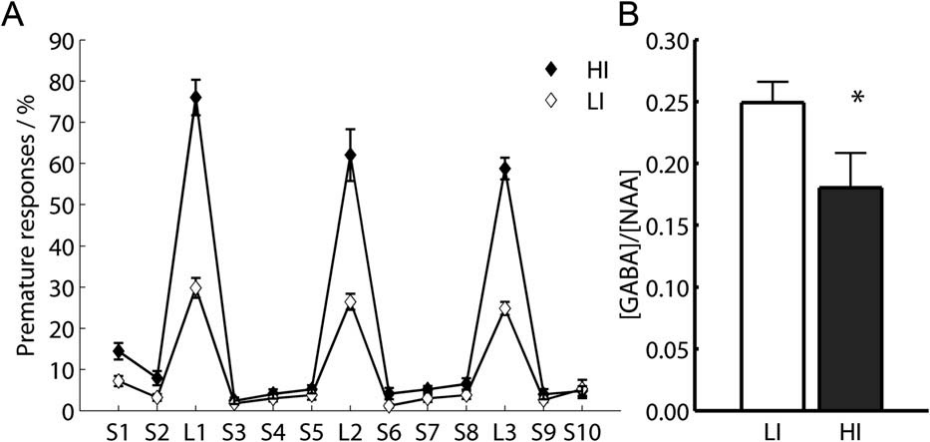
Impulsivity scores measured in rats **(A)** showing stability of high premature responding rates over successive long intertrial interval challenges (L1, L2, and L3) against successive baseline measures (S1, S2, …, S10). **(B)** GABA to NAA ratios for low‐impulsive (LI) and high‐impulsive (HI) rats. Asterisk indicates significance at *P* < 0.05 and error bars show 1 SEM.

Figure 5A shows the screening data used to segregate LI and HI rats according to the number of premature responses elicited during the three challenge sessions involving an extended waiting interval between the start of the trial and subsequent presentation of the light stimulus. It can be seen that HI rats made consistently more premature responses than LI rats across each of the three challenge days. Group‐averaged concentration ratios for LI and HI rats are shown in Fig. 5B, with HI rats exhibiting a 28% lower GABA/NAA ratio compared to LI rats (*P* = 0.02; 95% confidence interval [CI] [–53%, –2%]). Across all rats, the mean GABA to NAA ratio was 0.22 (95% CI [0.18, 0.25]).

To demonstrate that our findings were not confounded by variation in NAA concentration, we also calculated GABA/Cho and GABA/Cr ratios, which revealed the same significant contrast between HI and LI rats, specifically a 23% lower GABA/Cho ratio in HI rats (*P* = 0.03; 95% CI [–47%, 0%] and a 26% lower GABA/Cr ratio (*P* = 0.03; 95% CI [–52%, 0%]).

## Discussion

MEGA‐PRESS spectra obtained were of high quality from each rat, showing the feasibility of the method in rats at 4.7T.

We chose NAA to normalize the GABA signal for the least error, as it has the strongest signal in the water‐suppressed spectra. NAA is usually considered a neuronal marker, and as such is a better normalizing metabolite than water, in this case due to the likelihood of substantial variability in the fraction of ventricle present in each voxel. This is particularly important for this study due to the impact of variation in ventricle size and anatomy between rats with close proximity to the large voxel used. Small anatomical variations or errors in voxel placement could substantially increase the water signal, leading to underestimation of metabolite concentrations. Furthermore, the NAA signal is derived from the MEGA‐PRESS data and not from a separate water reference scan and so may be less susceptible to drift or motion occurring over time. To guard against misinterpretation due to NAA changes themselves, we calculated ratios based on creatine and choline and found no substantial difference in the results.

A criticism of the MEGA‐PRESS technique is the possibility of coedited macromolecule signals that can have a substantial contribution to the measured GABA signal,^13^ arising from spins at 3.0 ppm, which are coupled at 1.7 ppm and overlapping with the measured GABA peak. It has been suggested that this can be addressed by metabolite‐nulled spectra (eg, with an inversion time of 1 sec before acquisition)^14^, ^15^ but that was not attempted here. There is some evidence that metabolite‐nulling reduces the reliability of GABA measurements as it introduces sensitivity to motion and instabilities, as the nulled baseline is not acquired in an interleaved fashion with the MEGA‐PRESS spectra.^16^ Despite this, we cannot be certain that changes in macromolecule concentrations do not contribute to the differences observed here. As GABA is produced from glutamate by glutamate decarboxylase (GAD_65/67_), and this has been shown to be decreased in the ventral striatum of HI rats,^9^ it is plausible that the lower signals in HI rats were due to lower levels of endogenous GABA.

The measures of GABA concentration in rat brain found here compare well with other MRS measurements made using ^13^C doping (∼2.0 mM^17^ at 11.7T), ^1^H/^31^P (1.9 ± 0.4 mM^14^ at 8.4T). From studies primarily of rat cortex, values measured by selective homonuclear polarization transfer at 11.7T^18^ are closer to the values found in human brain, 1.2 ± 0.1 mM, with an ex vivo ^13^C value of 1.26 mM found previously.^19^ The slightly larger values implied by greater concentration ratios found in the present study may be a reflection of the larger proportion (∼95%) of GABAergic neurons of the rodent striatum^20^ or contamination by coedited macromolecules.

Negative associations of GABA concentrations measured by MEGA‐PRESS have been shown cortically in human studies of impulsivity^21^ (see Ref. 22 or a more comprehensive review of ^1^H‐MRS findings in neuropsychiatric conditions). These common findings reinforce the value of the translational methodology used here.

The limitations of this study are the ambiguity of GABA signals with coedited macromolecules and the relatively large voxel size compared to the anatomy of the small structures known to be involved in the neurobiology of impulsivity. Furthermore, as concentration ratios rather than absolute GABA quantification was used, it is possible that increases in other metabolites could explain the smaller ratios obtained in HI rats. Although a significant reduction in GABA ratios was shown in highly impulsive animals, there is still some overlap in the values measured in weakly impulsive animals, indicating that the GABA deficits alone are not a sufficient determinant of impulsive behavior.

In conclusion, we have demonstrated that MEGA‐PRESS in rats is achievable in a reasonable time giving high‐quality spectra, and further that GABA concentration ratios are significantly reduced in the ventral striatum of highly impulsive rats compared with weakly impulsive rats. It is possible that these findings can be used as the basis of a translatable biomarker to investigate the brain mechanisms of attention deficit / hyperactivity disorder, addiction, and other disorders of impulse control in humans.

## References

[1] Bowery NG , Smart TG . GABA and glycine as neurotransmitters: a brief history. Br J Pharmacol 2006;147(Suppl 1):S109–119. 16402094 10.1038/sj.bjp.0706443 PMC1760744

[2] Baulac S , Huberfeld G , Gourfinkel‐An I , et al. First genetic evidence of GABA(A) receptor dysfunction in epilepsy: a mutation in the gamma2‐subunit gene. Nat Genet 2001;28:46–48. 11326274 10.1038/ng0501-46

[3] Rossor MN , Garrett NJ , Johnson AL , Mountjoy CQ , Roth M , Iversen LL . A post‐mortem study of the cholinergic and GABA systems in senile dementia. Brain 1982;105(Pt 2):313–330. 7082992 10.1093/brain/105.2.313

[4] Lewis DA , Hashimoto T , Volk DW . Cortical inhibitory neurons and schizophrenia. Nat Rev Neurosci 2005;6:312–324. 15803162 10.1038/nrn1648

[5] Puts NA , Edden RA . In vivo magnetic resonance spectroscopy of GABA: a methodological review. Prog Nucl Magn Reson Spectrosc 2012;60:29–41. 22293397 10.1016/j.pnmrs.2011.06.001 PMC3383792

[6] Mescher M , Merkle H , Kirsch J , Garwood M , Gruetter R . Simultaneous in vivo spectral editing and water suppression. NMR Biomed 1998;11:266–272. 9802468 10.1002/(sici)1099-1492(199810)11:6<266::aid-nbm530>3.0.co;2-j

[7] Edden RA , Barker PB . Spatial effects in the detection of gamma‐aminobutyric acid: improved sensitivity at high fields using inner volume saturation. Magn Reson Med 2007;58:1276–1282. 17969062 10.1002/mrm.21383

[8] Waddell KW , Avison MJ , Joers JM , Gore JC . A practical guide to robust detection of GABA in human brain by J‐difference spectroscopy at 3 T using a standard volume coil. Magn Reson Imaging 2007;25:1032–1038. 17707165 10.1016/j.mri.2006.11.026 PMC2131736

[9] Caprioli D , Sawiak SJ , Merlo E , et al. Gamma aminobutyric acidergic and neuronal structural markers in the nucleus accumbens core underlie trait‐like impulsive behavior. Biol Psychiatry 2014;75:115–123. 23973096 10.1016/j.biopsych.2013.07.013 PMC3898085

[10] Robbins TW . The 5‐choice serial reaction time task: behavioral pharmacology and functional neurochemistry. Psychopharmacology 2002;163:362–380. 12373437 10.1007/s00213-002-1154-7

[11] Dalley JW , Fryer TD , Brichard L , et al. Nucleus accumbens D2/3 receptors predict trait impulsivity and cocaine reinforcement. Science 2007;315:1267–1270. 17332411 10.1126/science.1137073 PMC1892797

[12] Tkac I , Starcuk Z , Choi IY , Gruetter R . In vivo 1H NMR spectroscopy of rat brain at 1 ms echo time. Magn Reson Med 1999;41:649–656. 10332839 10.1002/(sici)1522-2594(199904)41:4<649::aid-mrm2>3.0.co;2-g

[13] Mullins PG , McGonigle DJ , O'Gorman RL , et al. Current practice in the use of MEGA‐PRESS spectroscopy for the detection of GABA. Neuroimage 2014;86:43–52. 23246994 10.1016/j.neuroimage.2012.12.004 PMC3825742

[14] Behar KL , Boehm D . Measurement of GABA following GABA‐transaminase inhibition by gabaculine: a 1H and 31P NMR spectroscopic study of rat brain in vivo. Magn Reson Med 1994;31:660–667. 7914662 10.1002/mrm.1910310612

[15] Hofmann L , Slotboom J , Boesch C , Kreis R . Characterization of the macromolecule baseline in localized (1)H‐MR spectra of human brain. Magn Reson Med 2001;46:855–863. 11675635 10.1002/mrm.1269

[16] O'Gorman RL , Edden RA , Michels L , Murdoch JB , Martin E . Precision and reliability of in vivo GABA and glutamate quantification. In: Proc 19th Annual Meeting ISMRM, Montreal; 2011 p 3434.

[17] Yang J , Johnson C , Shen J . Detection of reduced GABA synthesis following inhibition of GABA transaminase using in vivo magnetic resonance signal of [13C]GABA C1. J Neurosci Methods 2009;182:236–243. 19540876 10.1016/j.jneumeth.2009.06.015 PMC2738992

[18] Chen Z , Silva AC , Yang J , Shen J . Elevated endogenous GABA level correlates with decreased fMRI signals in the rat brain during acute inhibition of GABA transaminase. J Neurosci Res 2005;79:383–391. 15619231 10.1002/jnr.20364

[19] Mason GF , Martin DL , Martin SB , et al. Decrease in GABA synthesis rate in rat cortex following GABA‐transaminase inhibition correlates with the decrease in GAD(67) protein. Brain Res 2001;914:81–91. 11578600 10.1016/s0006-8993(01)02778-0

[20] Tepper JM , Tecuapetla F , Koos T , Ibanez‐Sandoval O . Heterogeneity and diversity of striatal GABAergic interneurons. Front Neuroanat 2010;4:150. 21228905 10.3389/fnana.2010.00150 PMC3016690

[21] Edden RA , Crocetti D , Zhu H , Gilbert DL , Mostofsky SH . Reduced GABA concentration in attention‐deficit/hyperactivity disorder. Arch Gen Psychiatry 2012;69:750–753. 22752239 10.1001/archgenpsychiatry.2011.2280 PMC3970207

[22] Ende G . Proton magnetic resonance spectroscopy: relevance of glutamate and GABA to neuropsychology. Neuropsychol Rev 2015;25:315–325. 26264407 10.1007/s11065-015-9295-8

